# RNAi as an emerging approach to control Fusarium head blight disease and mycotoxin contamination in cereals

**DOI:** 10.1002/ps.4748

**Published:** 2017-11-22

**Authors:** Ana Karla Machado, Neil A Brown, Martin Urban, Kostya Kanyuka, Kim E Hammond‐Kosack

**Affiliations:** ^1^ Department of Biointeractions and Crop Protection Rothamsted Research Harpenden UK; ^2^ Department of Biology & Biochemistry University of Bath, Claverton Down Bath UK

**Keywords:** wheat, barley, maize, Fusarium graminearum, host‐induced gene silencing (HIGS), spray‐induced gene silencing (SIGS), disease resistance, transgenic plants, deoxynivalenol, fungal diseases

## Abstract

Fusarium graminearum is a major fungal pathogen of cereals worldwide, causing seedling, stem base and floral diseases, including Fusarium head blight (FHB). In addition to yield and quality losses, FHB contaminates cereal grain with mycotoxins, including deoxynivalenol, which are harmful to human, animal and ecosystem health. Currently, FHB control is only partially effective due to several intractable problems. RNA interference (RNAi) is a natural mechanism that regulates gene expression. RNAi has been exploited in the development of new genomic tools that allow the targeted silencing of genes of interest in many eukaryotes. Host‐induced gene silencing (HIGS) is a transgenic technology used to silence fungal genes in planta during attempted infection and thereby reduces disease levels. HIGS relies on the host plant's ability to produce mobile small interfering RNA molecules, generated from long double‐stranded RNA, which are complementary to targeted fungal genes. These molecules are transferred from the plant to invading fungi via an uncharacterised mechanism, to cause gene silencing. Here, we describe recent advances in RNAi‐mediated control of plant pathogenic fungi, highlighting the key advantages and disadvantages. We then discuss the developments and implications of combining HIGS with other methods of disease control. © 2017 The Authors. *Pest Management Science* published by John Wiley & Sons Ltd on behalf of Society of Chemical Industry.

## 1 INTRODUCTION

Fusarium head blight (FHB) is a major fungal disease of multiple cereal crops, including wheat, barley, maize oat, rye and triticale. FHB causes significant yield losses, reduces grain quality and contaminates the grain with fungal mycotoxins, which are harmful to human, animal and ecosystem health. Since the 1980s, FHB disease has re‐emerged, resulting in various epidemic and pandemic events that coincided with changes in cultural practices, such as reduced stubble burning and the use of non‐tillage, which can increase disease pressure, and in climatic alterations favouring warm and humid weather conditions at crop anthesis.^1^

FHB is a global threat causing losses estimated at US $3 billion in the USA between the early 1990s and 2008.^2^ In 2012, the UK wheat harvest dropped by 13% compared with the previous year; this was attributed to a wet autumn followed by cold spring, which was favourable for the emergence of many diseases, including FHB.^3^ In addition, FHB epidemics are very recurrent in the developing world. In China, FHB is endemic in some regions, causing annually severe or moderate epidemics.^4^ In southern Brazil, where 90% of Brazilian wheat is grown, FHB caused losses ranging from 11.6% to 39.8% between 2000 and 2010.^5^

FHB disease is primarily caused by the ascomycete fungus *Fusarium graminearum*, and to a lesser extent, by other *Fusarium* species, namely *F. culmorum*, *F. pseudograminearum*, *F. avenaceum*, *F. poae*, other species belonging to the *F. graminearum* species complex and by some *Microdochium* species, such as *M. nivale*.^6^ Within plant tissue, *F. graminearum* can produce type B trichothecene mycotoxins, including deoxynivalenol (DON) and its acetylated derivatives 3‐acetyl and 15‐acetyl deoxynivalenol (3‐ADON and 15‐ADON), plus nivalenol. Consequently, many countries have established maximum permitted levels for the most prevalent *Fusarium* mycotoxins in cereals and cereal products, protecting consumers from mycotoxicosis.^7^

In this review, we highlight the inadequacies in current FHB control strategies and discuss the use of RNA interference (RNAi) as a potential new approach to control FHB and mycotoxin contamination. We review the recent studies and mechanisms underlying RNAi in filamentous fungal plant pathogens. Finally, we discuss the advantages and disadvantages of applying this technique in FHB disease management.

## 2 CURRENT FHB CONTROL STRATEGIES ON WHEAT

Multiple control strategies, including cultural practices, irrigation management, chemical control and genetic resistance, have been adopted to curtail the impact of FHB on small grain cereal production. Plant‐mediated genetic resistance to FHB represents the most cost‐effective control strategy.^8^ However, breeding for resistance to FHB and DON accumulation has proven to be slow and complex. To date, only a few moderately resistant wheat and barley cultivars exist, and inheritance of these traits is controlled by multiple quantitative trait loci (QTLs) and affected by environmental factors such as relative humidity, rainfall and temperature.^9^ In wheat breeding programmes, the Chinese cultivar Sumai‐3 is the most notable source of FHB resistance. Genetic analyses identified multiple QTLs responsible for Sumai‐3‐mediated FHB resistance. The major 3BS QTL, named *Fhb1*, provides resistance to the spread of infection throughout the wheat head and resistance to DON through detoxification to DON‐3‐O‐glucoside.^10^ *Fhb1* has been incorporated into many commercial cultivars, especially in China. To date, more than 50 QTLs for FHB resistance have been described from wheat genotypes other than Sumai‐3, but despite considerable efforts to breed FHB‐resistant cultivars, at present only moderate resistance to the spread of infection beyond the initially infected spikelet can be achieved. Under FHB favourable conditions, multiple infection events can occur and mycotoxin contamination of the grain remains an issue.^6^ Resistance to FHB in barley is even more complex and only a few QTLs have been identified with a small effect on FHB severity and DON concentration.^11^

Fungicides are an integral part of the FHB disease management strategy. Demethylation inhibitors (DMI) are the most common class of fungicides used to protect against FHB.^6^ These fungicides include the triazoles targeting one specific enzyme, cytochrome P450 lanosterol C‐14α‐demethylase (CYP51), which plays a key role in biosynthesis of ergosterol, an important component of the fungal cell membrane that mediates cell permeability and is essential for fungal growth and virulence.^12^ Although DMI fungicides can reduce FHB infection, it is near impossible to achieve complete control. This is because *F. graminearum* has a high intrinsic level of resistance to triazoles compared with other pathogens due to the presence of an additional CYP51 gene.^13^ Moreover, to control FHB, fungicides must be applied to the emerged wheat heads prior to flowering, which is when the crop becomes vulnerable to FHB. Hence, in the field, it is extremely difficult to protect all the wheat heads within the crop canopy with a single spray because plants and tillers do not always flower synchronously.^8^ Therefore, during fungicide evaluations, various parameters are recorded to determine the efficacy of treatments using single or mixed chemicals. These evaluations include visual disease assessments (typically incidence of infected spikelets), amount of *Fusarium* DNA (typically trichothecene synthase (*Tri5*) gene), total DON (DON, 3‐ADON and 15‐ADON) concentrations, 1000‐grain weight, damaged kernel ratings and extrapolated final crop yield.^12^ Recently, a tebuconazole‐resistant, highly aggressive and toxigenic *F. graminearum* strain emerged in the USA, indicating the potential for the evolution of fungicide‐resistant populations.^14^ Therefore, complete control of FHB and mycotoxin contamination is not possible at present and combined efforts are needed to develop new integrated FHB control strategies.

## 3 RNA MECHANISMS

RNA interference (RNAi), or RNA‐silencing, is a post‐transcriptional gene silencing mechanism, involving small RNA molecules that leads to sequence‐specific mRNA degradation.^15^ RNAi is reported to occur in all four eukaryote kingdoms.^16^ RNAi is typically initiated by introduction of long double‐stranded RNAs (dsRNA) into the cell. Long dsRNAs can be produced in different ways, such as the replication of RNA from an RNA template (RNA viruses), by hybridisation of complementary RNA transcripts, or from single‐stranded RNAs containing complementary or near‐complementary inverted repeats separated by a short spacer sequence that can fold back on themselves to form a hairpin (hpRNA).^17^ These dsRNAs are then cleaved by the RNase‐III‐like Dicer protein into 20–25 bp RNA duplexes with two‐nucleotide 3′‐overhangs, known as small interfering RNAs (siRNAs). One strand of siRNA (the guide) is loaded into an RNA‐induced silencing complex (RISC), whereas the other strand (the passenger) is degraded. An RNase protein called Argonaute forms the catalytic centre of the RISC. RISC degrades target mRNAs that are nearly perfectly complementary to the loaded guide strand of siRNA.^18^

Fungal RNAi mechanisms were first identified in the saprotrophic species *Neurospora crassa* and termed ‘quelling’.^19^ Quelling is active in the vegetative phase of the *N. crassa* life cycle and is necessary to control transposons.^20^ The mechanism and the core RNAi machinery, including Dicer, Argonaute and RNA‐dependent RNA‐polymerases (RdRps), appear to be largely conserved in fungi,^21^ but some differences do exist. In *N. crassa* and several other fungi such as *Mucor circinelloides*, additional genes involved in RNAi have been identified, with production of siRNAs by Dicer‐independent pathways.^22^ Moreover, some fungal species can lack some components of, or the entire, RNAi machinery. These include the budding yeast *Saccharomyces cerevisiae* and the corn smut fungus *Ustilago maydis*.^23^

The RNAi pathway in *F. graminearum* consists of two Dicer proteins (FgDicer1 and FgDicer2), two Argonaute proteins (FgAgo1 and FgAgo2) and five RdRps (FgRdRp1–5).^24^ The Dicer‐dependent RNAi machinery regulates sexual perithecia development in *F. graminearum*, but is not involved in fungal growth, asexual conidiation, abiotic stress or disease formation.^24^, ^25^ However, FgAgo1 and FgDicer2 seem to play a critical role in silencing endogenous *F. graminearum* genes triggered by a hpRNA expressed from a transgene.^24^ This approach utilised a RNAi vector containing an intron sequence between two inversely oriented and self‐complementary target sequences, which when expressed, generate a dsRNA molecule with a hairpin structure.^26^

In plants, and some animals, locally initiated gene silencing can spread to other parts of the organisms, through systemic or cell‐to‐cell transport of the silencing signal. In plants, the silencing signal is suggested to be transmitted long range by the phloem, following source to sink dynamics. Short distance and long‐range cell‐to‐cell silencing signal movement may also occur symplastically through specialised connections between cells called plasmodesmata.^18^ Studies in *Arabidopsis thaliana* demonstrated that different RdRps are required for local and systemic silencing. Therefore, local and systemic RNA silencing pathways may be distinct.^27^ A question that remains to be fully investigated is, do both siRNAs and dsRNAs (i.e. silencing signals) move systemically and locally from cell to cell?

## 4 RNA AND TRANS‐KINGDOM GENE SILENCING

**Figure 1:**
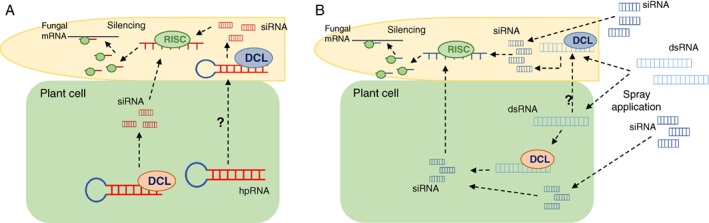
Possible pathways of host‐induced gene silencing (HIGS) and spray‐induced gene silencing (SIGS). (A) HIGS. Transgenic plant (introduction of transgenic hairpin RNA structure into plant genome). Long double‐stranded RNAs (dsRNAs) produced by the transgenic plant cells. These long dsRNAs could be cleaved into small interfering RNAs (siRNAs) by either the plant Dicer‐like proteins (DCL) or filamentous organism DCL proteins. Once plant siRNAs are present in the filamentous organism, the guide siRNA strand binds with Argonaute and other proteins to form a RNA‐induced silencing complex (RISC). The siRNA/RISC binds the complementary sequence of the target mRNA in the filamentous organism, resulting in degradation of the target transcript or inhibition of translation. (B) SIGS. Non‐transgenic organism (ectopic spray application of silencing molecules). External long dsRNAs and siRNAs are sprayed and can be taken up by both plant cells and filamentous organisms. The long dsRNAs in the plant cell could be processed into siRNAs by the plant DCL proteins or taken up by the filamentous organism. Long dsRNAs in the filamentous organism are processed into siRNA by the filamentous organism DCL protein. The guide siRNA strand binds to Argonaute and other proteins to form a RISC. The siRNA/RISC binds the complementary sequence of the target mRNA in the filamentous organism, resulting in degradation of the target transcript or inhibition of translation.

Since 2008, RNAi signals have been known to traverse between different organisms of the same or different species, and even across kingdoms, thereby providing another tier of communication, interaction and pathogen–host warfare. Both animal and plant host species exchange small RNAs with associated filamentous fungal or oomycete (protist) species, whether pathogenic or mutualistic.^28^, ^29^ A novel transgene‐based plant‐mediated approach was developed to produce siRNA that can silence gene transcripts in fungal and/or oomycete pathogens during infection, a process called host‐induced gene silencing (HIGS) (Fig. 1A). Researchers have hypothesised that the transport of siRNAs from the plant to the invading organism, such as a fungal pathogen, is mediated by exosomes (secreted vesicles), which are thought to be formed following the fusion of early secretion pathway‐derived vesicles (termed early endosome‐derived multivesicular bodies) with the plasma membrane.^30^ This hypothesis is supported by the fact that exosomes proliferate in plant cells during pathogen attack and are especially abundant when specialised pathogen–host interfaces form, for example, the extrahaustorial matrix.^31^ However, other mechanisms can be involved in the trafficking of siRNAs including passive diffusion, membrane‐associated transporters and receptors.^18^ Additional studies to verify these theories are necessary.

In filamentous fungi, HIGS was first demonstrated in 2010, through the silencing of a β*‐*glucuronidase (*GUS*) reporter gene in a transgenic strain of *F. verticillioides* during infection of transgenic tobacco plants expressing a hairpin *GUS* RNA.^28^ Subsequently, transgenic barley and wheat plants were engineered to express dsRNA targeting transcripts of the virulence factor *Avra10* in the fungus *Blumeria graminis*, which resulted in reduced powdery mildew infections.^29^ Numerous studies followed these seminal discoveries and these have revealed that HIGS is an effective approach to control a wide range of taxonomically unrelated filamentous fungal and oomycete pathogens.

RNAi can also occur naturally in the opposite direction, where filamentous organism‐induced gene silencing influences host plant target genes.^16^, ^32^ Even though it is well established that *Botrytis cinerea* has a necrotrophic *in planta* lifestyle, this fungus is now known to transfer small RNA ‘effectors’ into the cells of *Arabidopsis* and tomato plants.^32^ These fungal small RNAs originate from the long terminal repeat retrotransposons and are produced by the action of the fungal Dicer protein. The fungal small RNAs are capable of entering the plant cell where these molecules use the plant RNAi machinery, including the Argonaute proteins, to silence transcripts of plant genes involved in innate immunity and thereby to facilitate infection.^32^

An alternative non‐transgenic RNAi approach is spray‐induced gene silencing (SIGS), which exploits the RNAi mechanism, through the exogenous application of long dsRNA and siRNAs (Fig. 1B). SIGS was initially suggested and then used as a strategy to simulate HIGS, without the need to develop stably transformed plants.^33^ SIGS has since been demonstrated to be effective in controlling both *B. cinerea* and *F. graminearum*.^33^, ^34^ *Botrytis cinereal* mycelia growth *in vitro* can take up both external applied siRNAs and long dsRNA. Silencing the *B. cinerea* Dicer‐like 1 (*DCL1*) and *DCL2* genes by SIGS was therefore hypothesised to compromise filamentous organism‐induced gene silencing and lead to reduced disease. Indeed, an external spray application of siRNAs and long dsRNAs targeting fungal *DCL1* and *DCL2* to the surface of different fruits and vegetables, 3–5 days before inoculation with *B. cinerea*, significantly inhibited grey mould disease formation. Treatments carried out for rose petals, lettuce leaves, and fruits of tomato, strawberry and grape all led to reduction ranging from 60 to 80% in lesion size caused by *B. cinerea* compared with the three different types of control treatments, namely water, and yellow fluorescent protein gene‐specific either long dsRNAs or siRNAs.^34^ Although this study was not done using whole plants, the methods used demonstrate the potential applicability of SIGS technologies in multiple crop plant species. An overview of reported RNAi approaches from pathogenic ascomycete and basidiomycete fungi, the dsRNA delivery systems used, and the phenotypic outcomes of silencing observed are summarised in Table 1. By focusing on targeting fungal genes previously identified as being essential for pathogenesis, these approaches represent a promising technology and potentially a paradigm shift in crop protection. However, several challenges to its successful exploitation remain and these are discussed in Section 6.

**Table 1:** RNAi target genes tested in filamentous fungal plant pathogens using host‐induced gene silencing and spray‐induced gene silencing

Target species	Host	Target gene	Target gene function	Method	Phenotype	Ref
*Aspergillus flavus*	Maize	*aflR*	Aflatoxin biosynthesis transcription factor	HIGS^a^ (transgenic)	Transgenic plants accumulated lower levels of aflatoxins	47
*A. flavus* and *A. parasiticus*	Maize	*aflC*	Polyketide synthase (aflatoxin biosynthetic pathway)	HIGS (transgenic)	Aflatoxin was not detected in RNAi transgenic maize kernels	48
*Blumeria graminis*	Wheat Barley	*Avra10*	Virulence effector	BSMV–HIGS^b^ and HIGS (transgenic)	Reduced fungal development in the absence of host resistance gene *Mla10*	29
*B. graminis* f. sp*. hordei*	Barley	*BEC 1011* *BEC 1054* *BEC 1038* *BEC 1016* *BEC 1005* *BEC 1019* *BEC 1040* *BEC 1018*	Ribonuclease‐like protein Ribonuclease‐like protein Virulence effector Glucanase Metalloprotease Virulence effector Virulence effector	HIGS (transgenic)	Reduced virulence and reduced haustoria index	60 ^c^
*Botrytis cinerea* *V. dahliae*	*Arabidopsis* Tomato	*DCL1* *DCL2*	Dicer‐like protein Dicer‐like protein	SIGS^c^ HIGS (transgenic)	Reduced virulence	34
*Fusarium culmorum*	Wheat	*Fgl1* *Fmk1* *Gls1*	Secreted lipase Mitogen‐activated protein (MAP) kinase Beta 1,3‐Glucan synthase	BSMV–HIGS and HIGS (transgenic)	Reduced virulence	61
*F. graminearum*	*Arabidopsis* Barley	*CYP51*	Cytochrome P450 lanosterol C‐14α‐demethylase	HIGS (transgenic)	Reduced virulence	36
*F. graminearum*	Wheat	*Chs3b*	Chitin synthase 3b	HIGS (transgenic)	Reduced virulence	37
*F. graminearum*	Barley	*CYP51*	Cytochrome P450 lanosterol C‐14α‐demethylase	SIGS	Reduced virulence	33
*F. oxysporum* f. sp*. cubense*	Banana	*Velvet*	Transcription factor	HIGS (transgenic)	Reduced virulence	62
*F. oxysporum* f. sp. *conglutinans*	*Arabidopsis*	*FRP1* *FOW2* *OPR*	F‐box protein required for pathogenicity1 *F. oxysporum* wilt 2 12‐oxophytodienoate ‐10,11‐reductase	HIGS (transgenic)	Reduced virulence and delayed disease symptom development	45
*F. verticillioides*	Tobacco	*GUS* (*ß‐glucuronidase*)	Reporter	HIGS (transgenic)	Silencing of *GUS* transgene	28
*Puccinia striiformis* f. sp*. tritici*	Wheat	*PsCNA1* *PsCNB1*	Calcineurin homologue	BSMV–HIGS	Slower extension of fungal hyphae	63
*P. triticina*	Wheat	*MAPK1* *CYC1* *CNB*	Mitogen activated protein kinase Cyclophilin Calcineurin regulatory subunit	BSMV–HIGS	Reduced virulence	46
*Rhizoctonia solani*	Tall fescue	*RNApoly* *Imbs* *Coh* *UbiE3*	RNA polymerase Importin beta‐1 subunit Cohesin complex subunit Psm1 Ubiquitin E3 ligase	HIGS (transgenic)	Reduced virulence	64
*Sclerotinia sclerotiorum*	Tobacco	*Chs*	Chitin synthase	HIGS (transgenic)	Reduced virulence	65
*Verticillium dahliae*	*Arabidopsis* Tomato	*Ave1* *Sge1* *NLP1*	Virulence effector Transcription factor SIX gene expression Necrosis and ethylene‐inducing‐like protein	HIGS (transgenic)‐*Arabidopsis* TRV‐HIGS^d^ tomato	Reduced virulence *Sge1* in *Arabidopsis* *NLP1* in tomato and *Arabidopsis*	66
*V. dahliae*	Cotton	*VdH1*	Hydrophobin	HIGS (transgenic)	Reduced virulence	67

a HIGS (transgenic), host‐induced gene silencing in stable transgenic plants.

b BSMV–HIGS, BSMV‐mediated transient HIGS. *Barley stripe mosaic virus* is used as a vector for HIGS. The virus is inoculated in the host and siRNAs generated by the virus will be taken up by the fungal pathogen.^68^

In this study, 50 candidate effectors using HIGS were tested, but only the eight described above presented distinguished phenotype from the wild‐type.

c SIGS, spray‐induced gene silencing.^33^

d TRV‐HIGS, *Tobacco rattle virus* is used as a vector for HIGS. The virus is inoculated in the host and siRNAs generated by the virus will be taken up by the fungal pathogen.^69^

## 5 HIGS AND FUSARIUM HEAD BLIGHT

Several non‐conventional strategies that use advanced biotechnology to control FHB and reduce mycotoxin contamination, either directly or indirectly, have been explored. One of the most promising early successes was transgenic Bt maize, which has been developed to control European corn borer by producing *Bacillus thuringiensis* (Bt) toxin poisonous to this and related insect pests. Insects act as wounding agents and vectors spreading fungal spores to the plants, playing an important role in *Fusarium* infection in maize. The majority of studies carried out with transgenic Bt maize demonstrated that these plants were also less contaminated with *Fusarium* mycotoxins, including fumonisin, DON and zearelone than non‐Bt maize.^35^ A more recent strategy includes direct RNAi approaches, such as HIGS and SIGS, that have successfully silenced essential fungal genes and/or essential biosynthetic pathways.^33^, ^34^, ^36^, ^37^ Although *CYP51* is found in most eukaryotic organisms, the average nucleotide identity between *CYP51* genes from different species is very low (25–30%). This assisted in the design of HIGS constructs that could target just a single pathogen species. The use of HIGS to control *F. graminearum* was first demonstrated in 2013 under controlled environmental conditions, by silencing all three *CYP51* genes, namely *CYP51A, CYP51B* and the *Fusarium*‐specific CYP51C.^36^ CYP51 is the major target of azole fungicides, also known as DMIs (described in Section 2).^38^ Silencing *F. graminearum CYP51* genes *in vitro*, through the exogenous application of a 791‐nucleotide dsRNA complementary to each of the three *CYP51* paralogs, inhibited fungal growth and caused the abnormal branching of developing hyphae. Moreover, detached leaves of both transgenic *Arabidopsis* and barley plants expressing the same dsRNA were more resistant to *F. graminearum* infection compared with wild‐type plants,^36^ demonstrating the capacity of HIGS to silence fungal genes and impede infection.

In a follow‐on study, direct spray applications of the same 791‐nucleotide dsRNA onto detached barley leaves showed the potential for SIGS to silence the *CYP51* genes in *F. graminearum*, as described above for HIGS. Both, dsRNA‐treated and adjacent untreated leaf regions exhibited smaller lesions when infected with *F. graminearum* compared with leaves of non‐treated plants. These exogenously applied dsRNA moved through the phloem tissues and xylem in the plant vascular system. To demonstrate this movement, dsRNA labelled with a green fluorescent dye was sprayed onto the surface of detached barley leaves. In leaf cross‐sections, green fluorescence was observed in the xylem 24 h after spraying. Fluorescence in the symplast of phloem parenchyma cells, companion cells and mesophyll cells was also observed in longitudinal leaf sections.^33^ However, the use of detached leaves to apply the dsRNA could influence overall plant physiology and/or mobility of the silencing mechanism. Hence, the relevance of this approach under field conditions is not known. Previous *Arabidopsis* studies, which did not involve trans‐kingdom gene silencing, showed that siRNAs were mobile and triggered silencing in distant tissues.^39^ Therefore, both siRNA and long dsRNA may be mobile silencing signals, whereas differences in the mobility of distinct dsRNA species may depend on the organism, tissue analysed and/or method of delivery.

The bioassays used in these two *CYP51* gene silencing studies were primarily based on pathosystems involving either model host plant species (i.e. *Arabidopsis*) or tissues that do not represent natural *F. graminearum* floral infections (i.e. detached leaves).^33^, ^36^ Nonetheless, the reduction of *F. graminearum* infection achieved through the silencing of *CYP51* did provide novel mechanistic insights, while demonstrating that both the HIGS and SIGS can be used to silence *F. graminearum* genes which influence the outcome of infection.

In 2015, HIGS was reported to confer resistance to both seedling blight and FHB disease in intact wheat plants using artificial inoculations under controlled environmental conditions and following natural field infections.^37^ The wheat plants expressed HIGS constructs targeting the chitin biosynthesis pathway in *F. graminearum*. Chitin is an essential component of fungal cell wall and is synthesised by chitin synthase enzymes. Plant pathogenic fungi have numerous chitin synthase‐encoding genes.^40^ For example, the *F. graminearum* genome is predicted to contain eight chitin synthase genes, named *Chs1*, *Chs2*, *Chs3a*, *Chs3b*, *Chs4*, *Chs5*, *Chs6* and *Chs7*. Among these, *Chs3b*, showed the highest expression level during infection of wheat heads. Moreover, deletion of this gene in the fungus appeared to be lethal.^37^ For these reasons, *Chs3b* was selected as the target for HIGS. Three hairpin RNAi constructs, each targeting a different region in *Chs3b*, were co‐expressed as transgenes in the FHB‐moderate susceptible elite Chinese wheat cv. Yangmai 15. The resulting transgenic lines showed resistance to the spread of infection in the stem base at the young seedling stage, and in mature floral tissues at the adult plant stage consistently throughout the T_3_ to T_5_ generations. In the field, these transgenic RNAi lines exhibited a reduced number of *F. graminearum* infected spikelets. The reduction from 28–30% infected spikelets in the control plants to 7–11% in the two tested transgenic lines was comparable with the 7–8% infection in the moderate‐resistant wheat variety Sumai‐3. Additionally, a similar reduction in mycotoxin accumulation in grain was evident in the transgenic lines and in wild‐type Sumai‐3 (1.7–2.4 μg DON/g in the two test transgenic lines and 1.8 μg DON/g in Sumai‐3 compared with 11 μg DON/g in the control line).^37^ Therefore, silencing of *Chs3b* led to considerable DON reduction in single‐floret inoculations and natural field infections. However, the levels of DON detected were still above the maximum permitted limit in many countries.^7^ In that study, only visible disease symptoms were rigorously assessed. Ideally, the additional quantification and comparison of 1000‐grain weight, fungal biomass (*Tri5* DNA levels) and damaged kernel levels would have been informative. This approach is now common practice when evaluating and comparing the efficacy of single and multiple applications of different fungicides (as described Section 2).^12^ This would also ascertain whether the HIGS approach caused any yield penalty in grain production and/or had any adverse effects on plant physiology or plant development.

## 6 CHALLENGES AND BENEFITS OF USING SIGS AND HIGS TO CONTROL FHB AND OTHER DISEASES

The studies discussed above suggest that HIGS and SIGS could represent powerful approaches to control FHB and other fungal incited diseases. One immediate benefit is that the application of SIGS would overcome the issue of transgenic acceptance by the public presented by HIGS. However, some technical challenges remain that may hinder the use of SIGS as a mainstream control strategy. The first is the possibility that the effect of a single SIGS application in the field may only last for a few days, in which case precise application timing would be critical for success. To overcome this issue, a recent study has explored the use of double‐layered hydroxide clay nanosheets loaded with dsRNA, which can persist up to 30 days on sprayed leaves.^41^ These nanoparticles, first described in 2006, have to date been explored primarily in human therapeutics.^42^ The positively charged nanosheets bind to negatively charged dsRNA. The nanoparticles then react with atmospheric CO_2_ and humidity forming carbonic acid, which facilitates the gradual release of dsRNA.^41^, ^43^ Currently, a multinational company is developing a new technology for RNAi spray application targeting varroa mites, which infect honeybees, but details of this mechanism have not been revealed.^44^

A second, but no less important challenge is the costs associated with manufacturing and applying SIGS compared with conventional fungicides, due to the expense of RNA synthesis. However, this scenario has started to change. New technologies are being developed that allow the cost‐efficient mass production of RNA for topical RNAi applications in agriculture, which aims to produce RNAs for less than US $2/g;^44^ however, industry cannot yet estimate if this will be cheaper than fungicide applications. Although the main issues regarding SIGS applications are progressing towards pragmatic solutions, details on how long these dsRNAs or siRNAs travel and persist in the plant remain unknown. Therefore, the application of SIGS to control FHB, or indeed any other floral disease, could also encounter the same difficulties as traditional fungicide applications, namely the difficulty to protect all the wheat heads, which frequently emerge and flower at different times, with a single SIGS application.

The advantages and disadvantages of adopting HIGS to control disease are given in Table 2. Some of these are discussed in greater detail. Based on recent studies, HIGS of pathogen‐specific deemed 'essential for life' genes could be an efficient strategy to control FHB, as well as other fungal diseases and pests.^37^, ^45^, ^46^ Broad spectrum control of multiple pathogens using a single approach is highly desirable. By carefully designing the sequences to be used for HIGS and targeting the same gene in different fungal species, there is a strong possibility that broad spectrum control could be achieved.^26^ Exploring the vast data sets of genomic and transcriptomic information during the initial construct design phase in any project should decrease the chances of off‐target silencing of unintended genes in the host plants, as well as in the beneficial plant‐associated organisms, such as mycorrhizas, rhizobia and biocontrol species, like *Trichoderma* species. As a example, a HIGS study conducted in maize to downregulate aflatoxin biosynthesis in *Aspergillus flavus* caused stunting and reduced kernel placement in transgenic plants, potentially due to off‐target silencing of other genes.^47^ However, a reduction of aflatoxin production in transgenic maize carrying a different RNAi cassette targeting another pathogen gene showed no morphological alterations.^48^ Alternatively, the design of multiple silencing constructs that target more than one gene, which could subsequently be used within a concatenated/stacked HIGS cassette, could confer control against multiple pathogens from a simply inherited single genetic locus within a breeding programme.

**Table 2:** Advantages and disadvantages of adopting host‐induced gene silencing (HIGS) to control plant diseases

Host‐induced gene silencing	
Advantages	Disadvantages
Avoids application of multiple fungicides.	Consumers' concerns about transgenic crops.^a^
Efficient transformation protocols are available for most of the world's important stable crops, including wheat, barley, rice, maize, potato, soybean, canola.	An efficient transformation protocols is not available for some crop species.^a^
RNAi is sequence specific and therefore is more specific than most fungicides.	RNAi to protect against multiple pathogenic species may require concatenation/stacking of the sequences to be silenced.
The targets sites of commercial fungicides overcome by subtle pathogen sequence mutations can still be used as the target sequences for RNAi, thereby helping to provide control of emerging fungicide resistant strains in field populations.	Potential instability of HIGS transgene.
RNAi targets can have a few sequence mismatches and the silencing is still effective. Potentially therefore, RNAi is more difficult for mutations to render this technology ineffective.	Potential silencing of off‐target genes in the plant could adversely affect crop growth, reproduction and yield.
How the RNAi signal is amplified and spread among aphid cells. A gene that shares nucleotide sequence similarity among two or more pathogens can be used as a target to control multiple diseases.	Potential silencing of off‐target genes in plant associated organisms may affect plant beneficial relationships.
Multiple ‘essential for life’ genes have already been identified and published for plant pathogenic species and these could be the first targets for RNAi.^26^	Not all fungal species may be targeted through HIGS. Some fungi species apparently lack the whole or most of the RNA silencing components in the genome.^70^
The increased overall availability of genomic and transcriptomic sequence information for plants, pathogens, plant‐associated organisms, humans, other animals and insects, means that potential off‐target problems can be thoroughly investigated, predicted and ranked during the construct design phase in all projects.	Some pathogenic species may already possess or could evolve suppressors of the silencing mechanism as a counter‐defence strategy.
Broad spectrum control of multiple pathogens could be developed by targeting several pathogen genes within a single concatenated/stacked HIGS cassette. This cassette would be simply inherited as a single genetic locus within a breeding programme.	Within natural pathogen populations, variation may already exist in the efficiency of HIGS and SIGS between strains, isolates and/ or races, resulting in the least controlled individuals increasing in abundance when the technologies are first used.
HIGS construct expression can be constitutive or inducible (e.g. by pathogens) and can also be engineered to be tissue specific (e.g. floral spikes and not leaves or roots).^26^	HIGS approaches are unlikely to function post harvest to combat infections occurring in dried seeds, leaves, fruits and/or root. This is because of low overall plant physiological and metabolic activities and therefore limited opportunities to initiate and then systemically propagate the underlying silencing mechanisms.
Small interfering RNA and double‐stranded RNA technologies do not produce heterologous proteins that could lead to concerns about allergies.	

a SIGS can be used in these cases.

A potential counter mechanism is that pathogens could overcome HIGS through the acquisition of a suppression system. RNAi suppression is well characterised in plant viruses and has been reported previously in bacteria species.^49^ More recently, RNAi suppressors have been identified in *Phytophthora* species. In these oomycetes, the suppressors are secreted effector proteins that are delivered into/taken up by the host cell by unknown mechanisms, where they inhibit the accumulation of plant siRNAs.^50^ Therefore, the possibility exists that filamentous fungi have either acquired, or will evolve, a similar suppression system and would be able to suppress the HIGS or SIGS mediated technologies. This possibility has not yet been explored in a plant pathogenic fungal species.

Although, some concerns over using HIGS remain, transgenic crops are still considered as the fastest adopted crop technology in the history of modern agriculture and are cultivated in areas where more than half of the world population resides.^51^ In 2016, 19 developing countries planted 54% (99.6 million hectares) of the global transgenic crops, while seven developed countries accounted for the remaining 46% (85.5 million hectares).^51^ The USA and Brazil remained the top two producers of transgenic crops, accounting for 39% and 27% of the planted transgenic crops, respectively. Globally, in 2016, the most planted transgenic crops included soybean, maize, cotton and canola. To date, no transgenic wheat and barley are grown commercially, although many field tests have been conducted.

Transgenic acceptance of HIGS could be supported by the fact that dsRNA is highly specific (having the potential to be single species specific) and transgenic crops expressing dsRNA would not produce heterologous proteins that could lead to concerns about allergies. Recently, the first plant‐incorporated protectant based on RNAi technology was approved by the US Environmental Protection Agency. The term plant‐incorporated protectant refers to transgenic plants able to produce pesticides themselves. This approved product is a transgenic maize plant expressing dsRNA targeting *Snf7*, an important housekeeping gene in corn rootworms (*Diabrotica* spp.), which is a major pest in the USA that has developed resistance to many chemical pesticides.^52^

## 7 RNA ON THE FARM

RNAi technology has emerged as a promising alternative to fungicides and the deployment of resistant plant cultivars. RNAi is sequence specific and allows the targeting of individual problematic species.^53^ This specificity may be especially useful when most pathogenic species within a region can already be successfully controlled by conventional methods, and only one pathogenic species regularly persists on farm that requires an alternative control solution. In this scenario, a well‐timed SIGS application would probably be the most useful way to protect otherwise successfully growing crops.

In many agricultural systems, the efficacy of fungicides has been reduced due to the emergence of mutant variants in the pathogen population that are moderately or highly resistant to the chemistry.^14^ These reduced efficacy scenarios are frequently encountered where the same chemical group has been used for many years and/or when multiple applications are made each season. This loss in fungicide efficacy typically results from three underlying causes, namely: (1) a small number of sequence changes in the gene coding for the target protein which often alter the fungicide binding pocket; (2) over‐expression of the target protein due to specific changes in the promoter sequence (frequently duplication events); or (3) second site mutations at other loci in the pathogen genome that alter either fungal metabolism or specific detoxification pathways and reduce the capacity of the applied fungicide to reach to target protein.^12^ In the case of target site mutations, of either the first or second type, the use of a RNAi approach to target the silencing of the gene using the remaining unaltered regions of the target sequence is a feasible option to control fungicide resistant strains. For example, *F. graminearum* strains resistant to DMI fungicides have been identified recently that possess variant target *CYP51* sequences.^14^ Testing the efficacy of the already available transgenic plants carrying *CYP51* silencing constructs and SIGS constructs against DMI fungicide‐resistant strains would be highly informative. For non‐target site resistance problems, once the pathogen loci involved have been identified, these sequences could also be targeted via an RNAi approach to control fungicide resistance strains in field populations.

An emerging public concern is the possibility that plant siRNA or dsRNA present in food could be taken up by humans and animals, and affect mammalian gene expression. Some studies have reported that siRNA could be delivered into mammalian systems via the digestive tract,^54^ whereas other studies have revealed that ingested plant siRNA could not be detected in mammalian gut.^55^, ^56^ The main problem with the studies that have concluded the existence of a mechanism to transport exogenous small RNAs from the mammalian gut to target tissues within the animal is the lack of independently corroborating data.^55^ In addition, for a plant‐expressed siRNA or dsRNA to influence mammalian gene expression patterns, a complex series of events would need to be completed successfully, whereas the most plausible scenario post ingestion is the partial or complete degradation of siRNA or dsRNA in the digestive tract, leading to instability/loss of the molecule and a lack of uptake. If the siRNAs or dsRNA remains intact, each molecule type would then need to be delivered to a target tissue in a sufficient quantity to activate RNAi, as well as have sufficient sequence complementarity with an mRNA transcript in the target cells.^57^ The probability of the full sequence of events occurring is very low. Additionally, in nature, plants are known to produce siRNAs, microRNA and dsRNAs throughout their own growth and development to regulate normal plant physiological processes. Therefore, humans and mammals have been ingesting exogenous siRNAs and dsRNAs from a wide array of plant sources for many tens of thousands of years.

To take the HIGS or SIGS approach onto farms, the effects of environmental conditions, soil type, irrigation regimes and overall growing conditions through the season, on RNAi efficacy would need to be explored in detail. To date, these types of experiments involving field trials have not been reported in the literature. Variations in the efficacy of silencing have already been encountered, even in different laboratories.^53^

One aspect of disease control in which HIGS approaches are unlikely to function is post harvest to combat infections occurring in dried seeds, leaves, fruits and/or roots.^58^ This is because of the low overall physiological and metabolic activities occurring in these dried plant tissues, and therefore the limited opportunities to initiate and then systemically propagate the trans‐kingdom silencing mechanisms. Although SIGS should be effective in controlling pathogen growth and colonisation post harvest.

## 8 OUTLOOK

RNAi has emerged as a promising new approach to control fungal plant diseases. RNAi is sequence specific and therefore permits the highly specific targeting of individual fungal species, or specific orders of fungal pathogens. This is preferential, and distinct, to broad‐acting chemical antifungal treatments that promote the evolution of resistance in targeted and non‐targeted fungal populations. One example is the association between the use of azole fungicides in agriculture and the rise of azole‐resistant *Aspergillus* species in a clinical setting.^59^ The use of both SIGS and HIGS on a commercial scale appears possible in the near future. Similar HIGS‐based approaches developed to control FHB in wheat may be developed and assessed for their efficacy to control other *Fusarium*‐incited diseases of other important crops, e.g. banana, tomato, lettuce and oil palm, or to control other problematic fungal diseases of wheat, i.e. wheat blast caused by the ascomycete fungus *Magnaporthe oryzae* (*Pyricularia oryzae*) or stem rust caused by *Puccinia graminis* f. sp. *tritici*. Field trialling of RNAi technologies has only started in the past 5 years, but there already appears to be an urgent need to ensure that a suite of standard assessment methods and standardised controls (fungicide treatments and the use of semi‐resistant cultivars) are included in each field trial and the raw data sets are placed in the public domain to ensure the different control strategies (fungicides, breeding and RNAi) can be accurately compared. Currently, this is not done and therefore comparisons across technologies presented in the literature are possible only rarely. With the increased interest in the use of RNAi for fungal disease control, a greater understanding of the genes and pathways controlling the phenomena of the trans‐kingdom RNAi will emerge. This new knowledge should then help to further optimise the construction, deployment and re‐use of HIGS multigene cassettes for the sustainable control of plant diseases.
